# Assessment of the Effect of Recruitment Maneuver on Lung Aeration Through Imaging Analysis in Invasively Ventilated Patients: A Systematic Review

**DOI:** 10.3389/fphys.2021.666941

**Published:** 2021-06-04

**Authors:** Charalampos Pierrakos, Marry R. Smit, Laura A. Hagens, Nanon F. L. Heijnen, Markus W. Hollmann, Marcus J. Schultz, Frederique Paulus, Lieuwe D. J. Bos

**Affiliations:** ^1^Department of Intensive Care, Amsterdam UMC, University of Amsterdam, Amsterdam, Netherlands; ^2^Department of Intensive Care, Brugmann University Hospital, Université Libre de Bruxelles, Brussels, Belgium; ^3^Department of Intensive Care, Maastricht UMC+, Maastricht, Netherlands; ^4^Laboratory of Experimental Intensive Care and Anesthesiology, Amsterdam UMC, University of Amsterdam, Amsterdam, Netherlands; ^5^Department of Anesthesiology, Amsterdam UMC, University of Amsterdam, Amsterdam, Netherlands; ^6^Mahidol–Oxford Tropical Medicine Research Unit (MORU), Mahidol University, Bangkok, Thailand; ^7^Nuffield Department of Medicine, University of Oxford, Oxford, United Kingdom; ^8^Department of Respiratory Medicine, Amsterdam UMC, University of Amsterdam, Amsterdam, Netherlands

**Keywords:** electrical impedance tomography, computed tomography, lung ultrasound, overdistention, recruitment maneuvers, ARDS

## Abstract

**Background:** Recruitment maneuvers (RMs) have heterogeneous effects on lung aeration and have adverse side effects. We aimed to identify morphological, anatomical, and functional imaging characteristics that might be used to predict the RMs on lung aeration in invasively ventilated patients.

**Methods:** We performed a systemic review. Studies included invasively ventilated patients who received an RM and in whom re-aeration was examined with chest computed tomography (CT), electrical impedance tomography (EIT), and lung ultrasound (LUS) were included.

**Results:** Twenty studies were identified. Different types of RMs were applied. The amount of re-aerated lung tissue after an RM was highly variable between patients in all studies, irrespective of the used imaging technique and the type of patients (ARDS or non-ARDS). Imaging findings suggesting a non-focal morphology (i.e., radiologic findings consistent with attenuations with diffuse or patchy loss of aeration) were associated with higher likelihood of recruitment and lower chance of overdistention than a focal morphology (i.e., radiological findings suggestive of lobar or segmental loss of aeration). This was independent of the used imaging technique but only observed in patients with ARDS. In patients without ARDS, the results were inconclusive.

**Conclusions:** ARDS patients with imaging findings suggestive of non-focal morphology show most re-aeration of previously consolidated lung tissue after RMs. The role of imaging techniques in predicting the effect of RMs on re-aeration in patients without ARDS remains uncertain.

## Introduction

A lung recruitment maneuver (RM) is a dynamic and transient increase in transpulmonary pressure aiming at (re-)opening collapsed lung parts and increasing end-expiratory lung volume (Lapinsky and Mehta, 2005). In theory, opening of collapsed or “non-aerated” lung areas decreases shunt, improving both oxygenation and removal of CO_2_ (Hedley-Whyte et al., 1964; Neumann et al., 1999). Furthermore, atelectatic areas might cause stress on, or deformation of, aerated regions, resulting in additional injury of lung parenchyma (Gattinoni et al., 2012). Accordingly, decreasing atelectatic areas with RM could protect the lungs, a strategy often referred to as the “open lung concept” (Hes, 2015).

The value of RMs without the use of any imaging monitoring is disputed, as, so far, clinical studies have failed to show benefit with regard to patient-centered outcomes—and even suggest harm (Cavalcanti et al., 2017). The absence of net benefit might be explained by the heterogeneity and unpredictable effects of RMs on lung aeration (Sahetya and Brower, 2017; Mancebo et al., 2019). The pressure threshold that should be overpassed during RMs to open atelectatic lung units is multifactorial and cannot be calculated precisely (Sahetya and Brower, 2017; Gattinoni et al., 2017). Furthermore, any increase in airways pressure will result in higher pressures in all lung parts, also those that are “open,” and these areas might be harmed by overdistention (Gattinoni et al., 2019). Thus, the benefit of RMs needs to be balanced between re-aeration and overdistention.

Changes in lung morphology indicative of re-aeration or overdistention can be estimated using lung imaging (Gattinoni et al., 2020). Various imaging techniques like chest computed tomography (CT), electrical impedance tomography (EIT), and lung ultrasound (LUS) have been suggested to be useful to evaluate lung morphology and function in an individual patient (Godet et al., 2015). We performed a systematic review to describe imaging-based methods to assess re-aeration after RMs in patients receiving invasive ventilation at the intensive care unit or the operating room. In this review, we focus on the variability of imaging-based method definitions and the clinical utility of baseline imaging characteristics.

## Methods

This protocol was designed in accordance with Preferred Reporting Items for Systematic Reviews and Meta-Analyses (PRISMA) guidelines (Liberati et al., 2009). The study protocol has been registered on PROSPERO (CRD42020188056).

### Eligibility Criteria

The PICO used to define eligibility criteria are the following: (1) *P* (population): invasive mechanical ventilation either in the intensive care unit (ICU) or the operating room (OR) with or without ARDS, (2) *I* (intervention): recruitment maneuver of any sort, (3) *C* (comparison): LUS and/or EIT and/or CT was used to evaluate re-aeration of previously consolidated lung tissue, (4) *O*: baseline image characteristics were reported and evaluated for their predictive value of recruitment.

Only original studies written in English were included, whereas animal studies, case reports, comments, letters, and studies that enrolled pediatric patients were not included.

### Information Sources and Search

We searched EMBASE using PubMed on December 15, 2020 using the following key words: *((“diagnostic imaging” [Subheading] OR (“diagnostic” [All Fields] AND “imaging” [All Fields]) OR “diagnostic imaging” [All Fields] OR “ultrasound” [All Fields] OR “ultrasonography” [MeSH Terms] OR “ultrasonography” [All Fields] OR “ultrasound” [All Fields] OR “ultrasonics” [MeSH Terms] OR “ultrasonics” [All Fields]) OR (“ct” [All Fields]) OR “computed tomography” [All Fields]) OR ((“IEEE Int Conf Electro Inf Technol” [Journal] OR “eit” [All Fields]) OR “(electrical impedance tomography” [All Fields])) AND ((“positive-pressure respiration” [MeSH Terms] OR (“positive-pressure” [All Fields] AND “respiration” [All Fields]) OR “positive-pressure respiration” [All Fields] OR “peep” [All Fields]) AND Recruitment [All Fields])*.

### Study Selection

The identified studies were assessed for inclusion criteria based on title and then on abstract. For all selected papers, the full text was read and discussed between two authors (CP and LB). Studies that fulfilled the inclusion criteria were included in this review.

### Data Collection

For each included study, we collected data related to patient characteristics and whether they referred to ARDS patients or not. The type of recruitment maneuver that was used was categorized as (a) sustained inflation, (b) sigh, (c) pressure-control ventilation, and (d) variable ventilation (Rocco et al., 2010). We recorded the criteria that were used to define a “responder” to recruitment and the baseline characteristics to identify factors that differentiate between “responders” and “non-responders.” For those studies including patients with ARDS, we documented whether authors classified patients as having “focal” (i.e., radiological attenuations with lobar or segmental distributions) or “non-focal” (i.e., radiological attenuation with diffuse or patchy distribution) abnormal lung morphology.

### Bias Assessment

The Quality Assessment of Diagnostic Accuracy Studies-2 (QUADAS-2) was used for the assessment of the methodologic quality of selected studies (Whiting, 2011). The four recommended domains (i.e., patient selection, index test, reference standard, and flow/timing) were assessed for low, high, or unclear risk of bias. As for the reference standard domain, CT was considered the “gold standard” for assessing lung re-aeration. Given the insufficient evidence to classify LUS or EIT as adequate reference tests to assess lung aeration, we considered the risk of bias to be high. Concerns regarding applicability for the first three domains were also assessed and scored as low, high, or unclear.

### Synthesis of Results

The following data were combined into a table: patient group that was studied, number of patients, type of recruitment and maximal airway pressure reached, assessment of re-aeration of lung tissue, and criteria to define “responder.” The main findings of the study regarding heterogeneity in re-aerated lung tissue and differences between “responders” and “non-responders” were also shown. We further synthesized the current evidence for heterogeneity and prediction of recruitment response in an overview table, stratified per imaging method that was used. Finally, we linked the morphological characteristics derived from different imaging techniques of responders and non-responders in an overview figure.

## Results

### Included Studies

**Figure 1:**
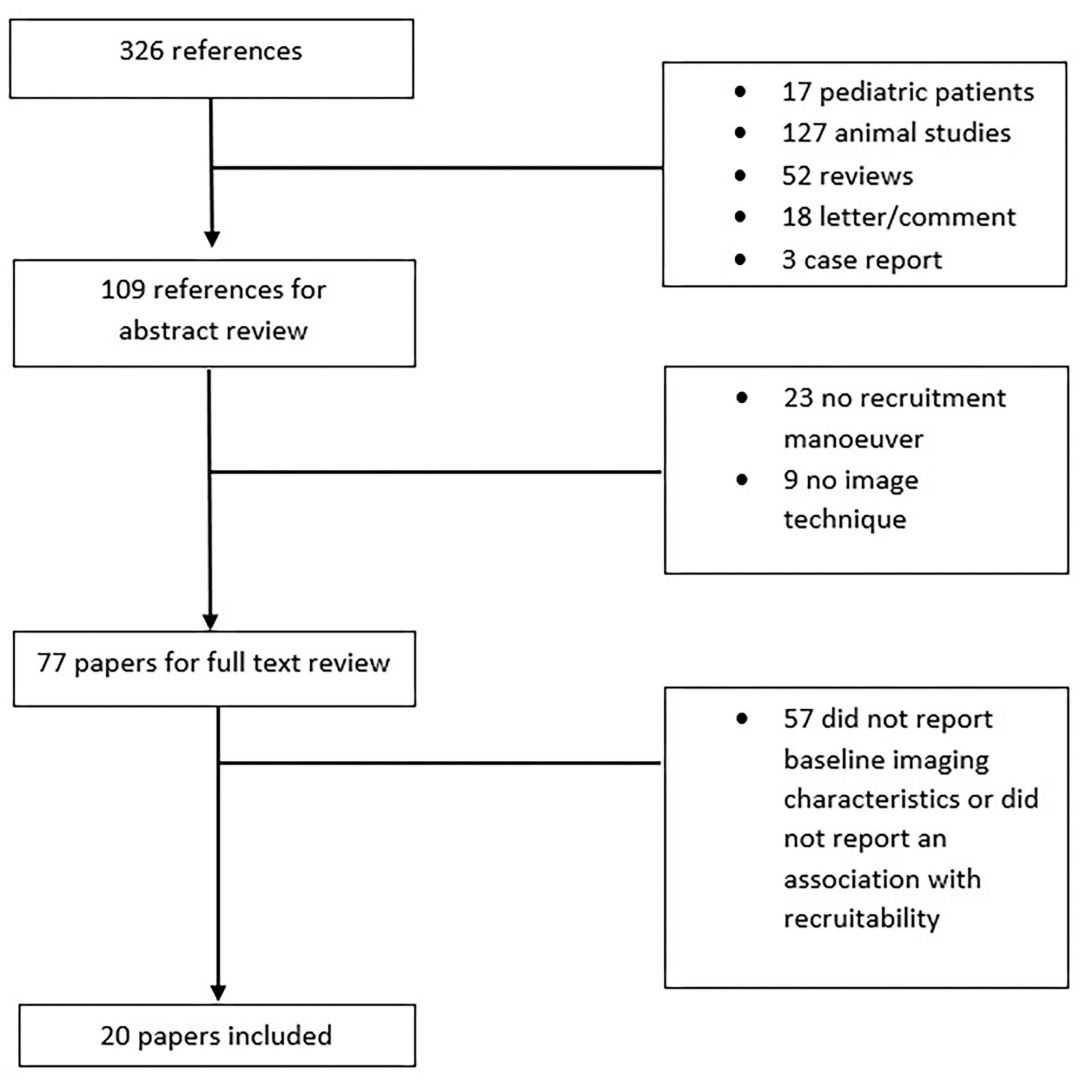
Flow diagram of the study selection.

The described search resulted in 326 articles of which 249 were excluded based on the title and abstract review. Twenty out of the remaining 77 studies were included in this review based on full text review (Figure 1) and are summarized in Table 1. Seventeen studies included deeply sedated patients, while sedation level was not mentioned in the other three studies. All patients in the included studies were in supine position during RM. The majority of the included studies enrolled ARDS patients exclusively (14 studies, 70%). Three studies (15%) included a mixed population of intensive care unit patients, and in three studies (15%), patients undergoing elective operation were included. Three studies had the primary goal of quantification of potential for lung recruitment (Gattinoni et al., 2006; Camporota et al., 2019) or recruitment prediction (Constantin et al., 2010). Regarding lung imaging techniques, most of the studies (10 studies, 50%) assessed chest CT scan, followed by LUS (five studies, 25%) and EIT (five studies, 25%). Notably, chest CT was only used in studies that included patients with ARDS.

**Table 1:** Studies included in this review.

**References**	**Patients**	**N**	**RM**	**Pmax**	**Imaging modality**	**Recruitment definition method**	**Outcome**
He et al., 2020	ICU (deeply sedated)	30	PC	NG	EIT	Ratio overdistended to recruited pixels	RM resulted in a high variability of the changes in the ration of overdistended to recruited pixels measured with EIT. No differences in the EELI and GI between responders and not responders to RM
Généreux et al., 2020	OR (deeply sedated)	45	SI	30 cm H_2_O	LUS	12 areas derived LUS score	RM did not result in a significant improvement in LUS score
Karsten et al., 2019	ICU (NM)	15	Sigh	40 cm H_2_O	EIT	Local compliance (ODCL index)	RM resulted in the complete disappearance of collapsed units (ODCLindex) in all studied patients, but there was a high variation of the overdistention extension (19 ± 17%). After RM, the proportion of collapsed units was highly variable (0–50%), independent of the selected PEEP (5–13 cm H_2_O)
Zhao et al., 2019	ARDS (deeply sedated)	3	Sigh	35 cm H_2_O	EIT	Increase in ventilation in dependent areas	Those with ventilation distribution predominantly in the most dependent regions are likely non-responders to RM
Camporota et al., 2019	ARDS (sedation level not mentioned)	47	SI	45 cm H_2_O	CT	Proportion of re-aerated lung tissue compared with the total lung weight	RM resulted in a variable change in aerated lung tissue with a mean of 24.3% (−2–76). All patients were on ECMO and had a very high percentage of non-aerated lung tissue. Non-recruitable tissue varied between 50 and 80% of total lung weight
Eichler et al., 2018	OR (deeply sedated)	37	Sigh	40 cm H_2_O	EIT	EELI slope	A downward course of EELI may indicate the need for RM (EELI_30sec_/EELI_0sec_ <1). This pattern of EELI inversed after RM and PEEP increase
Tang et al., 2017	ARDS (deeply sedated)	40	PC	35 cm H_2_O	LUS	Regasification score	RM resulted in significant changes in aeration in the anterior and lateral areas, but not in the posterior areas
Longo et al., 2017	OR (deeply sedated)	40	Sigh	35 cm H_2_O	LUS	Resolution of atelectasis	RM resolved atelectasis in all but 2/20 (10%) of the patients. The RM effect was assessed with TOE
Eronia et al., 2017	ICU (deeply sedated)	16	SI	40 cm H_2_O	EIT	EELI slope	A downward course of end-expiratory lung impedance may indicate the need for RM (10 min delta EELI >10%). This pattern of EELI inversed after RM and PEEP increase
Chiumello et al., 2016	ARDS (sedation level not mentioned)	22	Sigh	NG	CT	Proportion of re-aerated lung tissue compared with the total lung weight	Responders to RM (increase in tissue >-100 HU) had higher amount of non-inflated tissue at PEEP 5 cmH_2_O (*r*^2^ = 0.44). This relation disappears when responders are defined by increase in tissue >-500 HU (*r*^2^ = 0.002)
^*^Caironi et al., 2015	ARDS (deeply sedated)	14	PC	45 cm H_2_O	CT	Proportion of re-aerated lung tissue compared with the total lung weight	Responders to RM had higher total lung weights. RM results in a highly variable recruitment of non-aerated lung tissue. This is independent of the severity of disease and baseline PEEP
de Matos et al., 2012	ARDS (deeply sedated)	51	PC	60 cm H_2_O	CT	Sectional lung weight re-aerated	RM resulted in variable aeration of previously non-aerated lung tissue: 45% (25–53). Responders to RM did not have a higher initial amount of non-aerated tissue (PEEP 10 cmH_2_O; *r*^2^ = 0.03)
Rode et al., 2012	ARDS (deeply sedated)	17	Sigh	30 cm H_2_O	LUS	Crater-like consolidations' borders leveling and abutting pleural line	RM resolved most (92%) of crater–like subpleural consolidations visible during ZEEP
Bouhemad et al., 2011	ARDS (deeply sedated)	40	SI	40 cm H_2_O	LUS	Increase lung re-aeration score	RM was unlikely to affect consolidations in posterior and caudal regions. RM responders were more likely to have non-focal rather than focal lung morphology
Constantin et al., 2010	ARDS (deeply sedated)	19	SI	40 cm H_2_O	CT	Proportion of re-aerated lung volume compared with the total lung volume	RM responders were more likely to have non-focal than focal lung morphology at ZEEP. Hyperinflation during RM is predicted by the lung volume between −800 and −900 HU in ZEEP (*r*^2^ = 0.77)
^*^Caironi et al., 2010	ARDS (deeply sedated)	68	PC	45 cm H_2_O	CT	Proportion of re-aerated lung tissue compared with the total lung weight	RM responders had more opening and closing lung tissue at PEEP 5 cm H_2_O. RM responders had a homogeneous cephalo-caudal distribution of non-aerated areas, while non-responders had a linear cephalo-caudal increase in non-aerated areas
Gattinoni et al., 2006	ARDS (sedation level not mentioned)	68	PC	45 cm H_2_O	CT	Proportion of re-aerated lung tissue compared with the total lung weight	RM had a variable effect on opening of lung tissue (median 9% range −10–60%). RM response was predicted by recruitment of lung tissue after increase in PEEP from 5 to 15 cm H_2_O (*r*^2^ = 0.72). RM response was predicted by the amount of non-aerated tissue at PEEP 5 cm H_2_O
Borges et al., 2006	ARDS (deeply sedated)	26	PC	60 cm H_2_O	CT	Proportion of re-aerated lung tissue compared with the total lung weight and proportion of re-aerated lung volume compared with the total lung volume	RM shows different responses with variation in lung opening pressures. RM at 40 cmH2O resulted in response in <50%, while this increased to 93% at 60 cm H_2_O
^*^Nieszkowska et al., 2004	ARDS (sedation level not mentioned)	32	Sigh	NG	CT	Volume increase in non-aerated or poorly aerated areas	RM responders more frequently had non-focal morphology rather than focal (lobar) morphology (recruited volume: 572 ± 25 vs. 249 ± 159 ml). RM did not result in overinflation in patients with a diffuse morphology
Vieira et al., 1999	ARDS (sedation level not mentioned)	14	Sigh	45 cm H_2_O	CT	Total lung volume increases	RM responders more frequently had a non-focal morphology. RM responders more frequently had a biphasic lung density histogram with a peak at −700 to −900 HU >50 ml at ZEEP is related to a higher amount of overinflation with RM

*OR, operating room; N, number of enrolled patients; Pmax, maximum pressure used for recruitment maneuver; RM, lung recruitment maneuver; SI, sustained inflation; PC, pressure control; LUS, lung ultrasound; EIT, electrical impedance tomography; CT, computed tomography; ODCL, overdistention collapse index; PEEP, positive end-expiratory pressure; ZEEP, zero end-expiratory pressure; EELI, end expiratory lung impedance; LIL, left inferior lobe; TOE, transesophageal echocardiography; HU, Hounsfield units; COPD, chronic obstructive pulmonary disease*.

* *Retrospective study*.

Quality characteristics of the included studies, in relation to the aim of this systematic review, are presented in Supplementary Table 1. In two studies, there was a high concern regarding applicability of population selection. These two studies included a highly selective population, i.e., patients after cardiac surgery (Longo et al., 2017) or patients who underwent tracheostomy (Eichler et al., 2018).

### Recruitment Methodology and Identification of “Responders”

**Figure 2:**
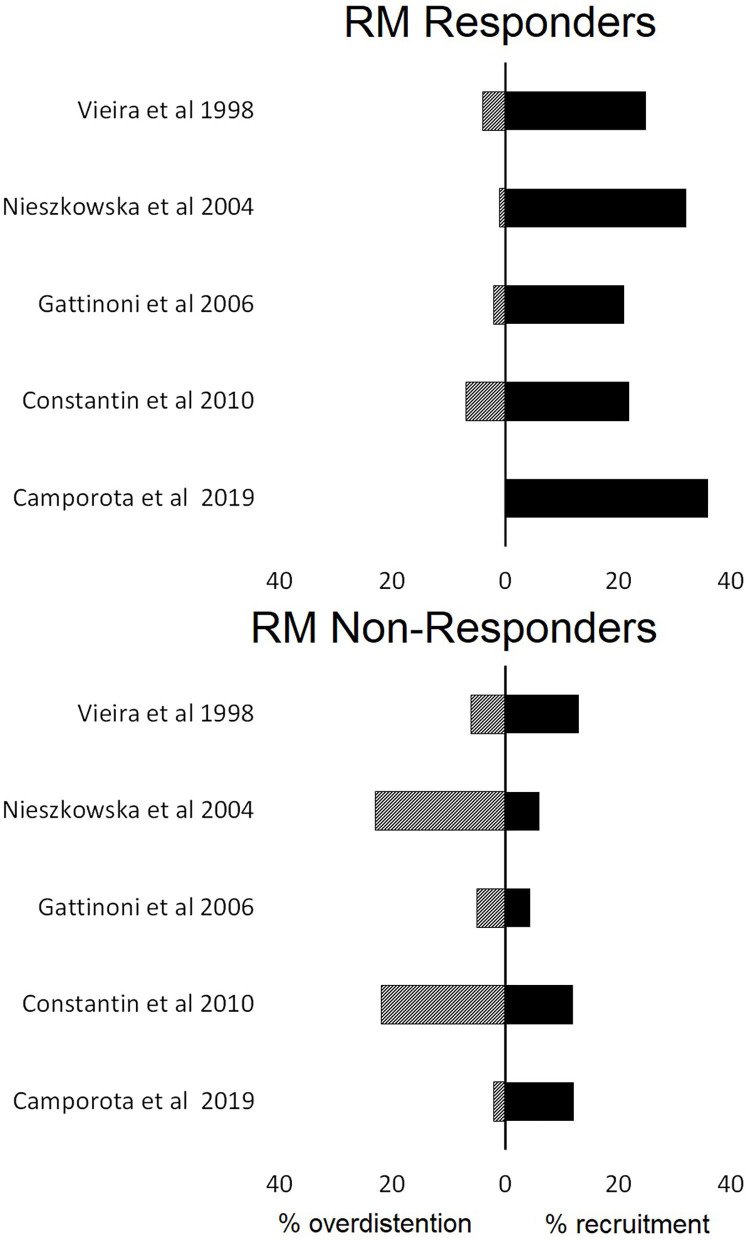
The proportions of lung recruitment and lung overdistention in patients who were characterized responders or not responders to lung recruitment maneuvers (RM) based on computed tomography findings.

In eight studies (42%), a sigh, in six studies (31%), a pressure-control method, and in five studies (26%), a sustained inflation were used for the RM (Table 1). Applied maximum airway pressure varied widely, between 30 and 60 cm H_2_O. Classification of responders depended on the imaging technique used (Table 2). None of the studies defined the criteria to identify “responders” beforehand. Patients were classified *post-hoc* as “responders” and “non-responders” based on the median value of the study population in studies that quantified re-aeration by CT imaging. Recruitment “responders” generally had an increase in aeration of non-aerated lung tissue of more than 20% (Figure 2).

**Table 2:** Findings related to the assessment of recruitment after recruitment maneuver application.

**Imaging modality**	**Definition of “recruitment”**	**Base-line PEEP**	**Maximum applied pressure (mean and range)**
LUS	Decrease four points in LUS score (Généreux et al., 2020)	ZEEP (Bouhemad et al., 2011; Rode et al., 2012; Tang et al., 2017; Généreux et al., 2020), 6 cm H_2_O (Longo et al., 2017)	34 cm H_2_O [30–40]
	Maximum increase in regasification score (Tang et al., 2017)		
	Disappearance of atelectasis or B-lines (Bouhemad et al., 2011; Rode et al., 2012; Longo et al., 2017)		
EIT	Any decrease in ODCLindex (Karsten et al., 2019)	ZEEP (Karsten et al., 2019; He et al., 2020), 5–8 cm H_2_O (Zhao et al., 2019), PEEP/FiO_2_ table PEEP(Eronia et al., 2017), 8 cm H_2_O (Eichler et al., 2018)	39 cm H_2_O [35–40]
	Reverse in EELI ratio from <1 to >1 (Eronia et al., 2017; Longo et al., 2017; Zhao et al., 2019)		
	Changes in the pixel ratio of overdistention to recruitment >15% (He et al., 2020)		
CT	Decrease in non-aerated weight of lung (>-100 HU) (Borges et al., 2006; Gattinoni et al., 2006; Caironi et al., 2010, 2015; de Matos et al., 2012; Chiumello et al., 2016; Camporota et al., 2019)	ZEEP (Vieira et al., 1999; Nieszkowska et al., 2004; Constantin et al., 2010), 5 cm H_2_O (Gattinoni et al., 2006; Constantin et al., 2010; Caironi et al., 2015; Chiumello et al., 2016; Camporota et al., 2019), 10 cm H_2_O (de Matos et al., 2012), 5–10 cm H_2_O (Borges et al., 2006)	48 cm H_2_O [40–60]
	Decrease in non-aerated and poorly aerated weight of lung (>-500 HU; Chiumello et al., 2016)		
	Increase in the volume of gas penetrating in non-aerated areas (>-500 HU; Borges et al., 2006)		
	Increase in the volume of gas penetrating in non-aerated and poorly aerated areas (>-500 HU; Vieira et al., 1999; Nieszkowska et al., 2004; Constantin et al., 2010)		

*PEEP, positive end-expiratory pressure; ZEEP, zero end-expiratory pressure; LUS, lung ultrasound; EIT, electrical impedance tomography; CT, computed tomography; EELI, end expiratory lung impedance; HU, Hounsfield units; ODCL, overdistention collapse index*.

### Heterogeneity in Re-aeration and Prediction of Positive Response to RM

Re-aeration after RM varied widely between studies, independent of the used image technique (Table 3). Unsurprisingly, most CT imaging studies showed that around 50% of patients are “non-responders” to recruitment because the median value was used as the cutoff value (Borges et al., 2006; Gattinoni et al., 2006; Caironi et al., 2015; Vieira et al., 1999; Camporota et al., 2019). Studies that used other imaging techniques did not mention the proportion of “non-responders,” though recruitment was described as “highly variable” (Karsten et al., 2019; Généreux et al., 2020).

**Table 3:** Observed recruitment maneuver re-aeration effect and findings related to potential for lung re-aeration after recruitment maneuver according to the imaging module and the presence or not ARDS.

	**ARDS**	**Non-ARDS**
**Observed lung re-aeration with imaging analysis**		
LUS	8% of evaluated consolidations did not respond to RM (Rode et al., 2012)	No change of LUS score after RM (Généreux et al., 2020)
	27% of patients had a re-aeration score ≥8 and an increase in lung volume more than 600 ml after RM (Bouhemad et al., 2011)	10% of patients do not respond to RM (Longo et al., 2017)
EIT	Extremely high variability in changes of the ratio between overdistention and collapsed ration (He et al., 2020)	Variable* compromise between the extension of lung collapse and overdistention after RM (Karsten et al., 2019)
CT	High variability* of potential recruitment tissue (Caironi et al., 2015)	
	Potential recruitable tissue: 45% (range 5–75%; de Matos et al., 2012)	
	Potential recruitable tissue: 9% (range −10–60%; Gattinoni et al., 2006)	
	Potential recruitable tissue: 24.3% (range −2–76; Camporota et al., 2019)	
	High variability of opening lung pressures (Caironi et al., 2015)	
**Findings that predicted more lung re-aeration**		
LUS	Anterior located consolidations (Bouhemad et al., 2011; Tang et al., 2017)	
	Crater-like sub-pleural consolidations (Rode et al., 2012)	
EIT	Predominant ventilation in non-dependent areas (Zhao et al., 2019)	Decreasing pattern of EELI (delta EELI >10% or EELI index <1; Eronia et al., 2017; Eichler et al., 2018)
CT	Not aerated tissue (>-100 HU) >25–30% of total lung tissue (Gattinoni et al., 2006; Chiumello et al., 2016)	
	Non-focal lung morphology (Nieszkowska et al., 2004; Constantin et al., 2010)	
	Homogeneous cephalo-caudal distribution of 40–50% non-aeration area (Caironi et al., 2010)	
	Opening and closing lung tissue (141 ± 81 g; Caironi et al., 2010)	

**Figure 3:**
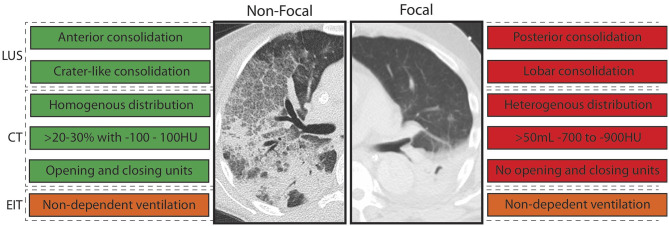
Imaging abnormalities that predicted response to recruitment maneuvers (RM) stratified per morphology. LUS, lung ultrasound; EIT, electrical impedance tomography; CT, computed tomography; HU, Houndsfield units; green, imaging abnormality in line with responder to RM; red, imaging abnormality in line with non-responder to RM; orange, imaging abnormality in line with responder with high uncertainty. Text boxes on the left: consistent with non-focal morphology. Text boxes on the right: consistent with focal morphology.

Imaging findings related to the amount of re-aerated lung tissue in patients with ARDS were the extent of lost aeration before RM, the distribution of non-aerated areas (craniocaudal and anteroposterior distribution), the morphology of non-aerated areas (e.g., crater-like consolidation), and functional lung characteristics related to tidal recruitment (tidal opening/closing tissue; Table 3). Findings that are more likely to resemble a diffuse or patchy loss of aeration (i.e., non-focal morphology) were suggestive of an increased likelihood of positive response to RMs (Figure 3). This was independent on the image technique employed.

Only one study addressed the prediction of response to RM in patients in the operating room. A decreasing pattern of end-expiratory lung impedance (EELI) evaluated with EIT was found to be related to the amount of re-aerated lung tissue (Eichler et al., 2018; Table 3).

### Overdistention

Overdistention was assessed in studies that used CT or EIT only, as LUS cannot be used for this purpose. Studies employing CT imaging showed the average percentage of overdistended lung volume to vary between 0 and 20% (Figure 2). EIT studies revealed the average overdistention secondary to RMs across patients to range between 5 and 30% (Karsten et al., 2019). Nevertheless, local overdistention in non-dependent areas may exceed 60% of that area (Eronia et al., 2017). “Non-responders” identified by CT had a higher increase in hyperinflated lung tissue compared with “responders” (Figure 2).

## Discussion

The results of this systematic review can be summarized as follows: (a) data that quantify the potential for lung recruitment based on imaging are limited, (b) the definition of positive response to RMs was highly variable, and (c) patients with imaging characteristics suggestive for a non-focal morphology of ARDS seemed to show more re-aeration at RMs with moderate inspiratory pressures.

The included studies used a wide range of maximum airway pressures to recruit lung tissue. Most collapsed areas can be opened, but frequently only at very high airway pressures (Cressoni et al., 2017). Borges et al. found opening pressures of 60 cmH_2_O in patients with ARDS to be common, with coexistence of areas opening at lower and higher pressures in the majority of patients (Borges et al., 2006). In clinical practice, maximum airway pressure is often selected based on the hemodynamic fragility of the patient rather than the expected pressure needed for lung recruitment (Santos et al., 2015). This might explain why CT compared with LUS and EIT studies revealed higher recruitment pressures as transfer for CT imaging requires more hemodynamically stable patients (Constantin et al., 2019). Recent RCTs suggest airway pressures above 50 cm H_2_O to be associated with serious adverse events, even when the patient is exposed to it for a short period of time (Cavalcanti et al., 2017; Hodgson et al., 2019). As the different components that attribute to the compliance of the respiratory system (compliance of the lung and chest wall as well as intra-abdominal pressure) cannot be easily separated in clinical practice (Umbrello and Chiumello, 2018), assessing the RMs' effect with imaging techniques is important in clinical practice. Rather than defining the pressure at which the lung can be opened, it is more important to determine whether recruitment can be achieved with moderate airway pressures. In other words, when comparing patients with a similar expected risk of side effects due to a transient increase in inspiratory pressures, a patient who responds to the RM with reaeration of previously collapsed lung tissue may still benefit, but a patient without this response may not.

This review also revealed several challenges associated with the quantification of lung *re-aeration* with image technics: there is poor agreement between imaging techniques, and there is no universal definition of recruitment response. Chiumello et al. found poor agreement between CT and LUS with respect to assessment of re-aeration, which is not unexpected since LUS is a semiquantitative method assessing only the subpleural areas (Chiumello et al., 2018). Furthermore, the role of LUS in assessing overdistention is currently unknown (Bouhemad et al., 2015). Pleural line displacement identified with LUS, as well the number of A-lines are relevant indexes that are currently being studied (Martins and Nogué, 2020; Tonelotto et al., 2020). EIT quantifies collapsed lung units based on local changes in compliance (Costa et al., 2009). However, compliance might be more related to the improvement or deterioration of already ventilated lung units than the real recruitment of atelectatic lung units (Chiumello et al., 2016). Even though CT is considered the gold standard in detecting lung recruitment, defining the degree of re-aeration remains challenging. Potentially recruitable lung tissue, determined by CT, is mainly expressed as percentage of total lung volume since absolute values depend on lung dimensions. However, expressing recruitment as percentage implies mathematical coupling with the total atelectatic volume, which is at least debatable (de Matos et al., 2012). Gattinoni et al. introduced the terms “high” and “low” recruitment responders based on the median percentage of potentially recruitable lung tissue determined by CT (Gattinoni et al., 2006). Worth mentioning, different median percentages of potentially recruitable tissue were reported in later studies (Camporota et al., 2019; de Matos et al., 2012), probably due to heterogeneity in inclusion characteristics and application of various maximum airway pressures. Given that recruitment is a continuous spectrum that depends on applied airway pressure and several imaging characteristics, speaking about “responders” from “non-responders” is a false dichotomization.

We set out to determine the role of imaging techniques in predicting the lung response to RM. The main strength of this review is the systematic and integrative approach. We excluded studies that based assessment of recruitment on mechanical or oxygenation variables as those can be influenced by factors other than recruitment of lung tissue, which is also known as the recruitment paradox (Amato and De Santis Santiago, 2016). We also acknowledge several limitations. First, we had to perform secondary analyses of many included studies as they were not intended to quantify potential for lung re-aeration, limiting statistical comparisons between groups. Second, we did not directly compare imaging techniques. Each method has intrinsic limitations, such as visualization of the subpleural region only for LUS and the need for patient transport for CT, which justify preferential use of one technique over another in specific situations. Of note, the definition and method of recruitment varied between studies even when the same image technique was used, which made direct comparisons impossible. Third, given the undefined role of LUS and EIT in the assessment of recruitment, a significant number of trials had an unclear risk of bias.

All features predictive of increased lung re-aeration after RM are consistent with a non-focal morphology of ARDS. Patients with focal ARDS lack, by definition, ventral consolidations not limited to the subpleural space and show a heterogeneous distribution of consolidation with less opening and closing, which renders them very unlikely to be recruitable. In line with this notion, patients with non-focal morphology were typically recruitable, while patients with focal morphology were not (Puybasset et al., 2000; Constantin et al., 2010). Notably, atelectasis is usually located in the dorsal lung areas in patients without lung injury requiring invasive mechanical ventilation (Longo et al., 2017; Pereira et al., 2018) implying a “focal” morphology. This may explain the lack of RM efficiency to increase lung aeration in invasively ventilated patients in the operating room (Généreux et al., 2020). Although the results of this review are not conclusive for patients without ARDS, it stresses the need for further research into lung morphology and its relation to lung re-aeration with robust imaging technics in these patients.

By integrating data from multiple studies to morphological classifications, we present a framework used to better design and interpret future studies. We have to acknowledge that this classification is imperfect, as one EIT study that only included three patients suggested that predominant ventilation in the non-dependent areas predicted recruitment, while this is not a feature that is consistent with non-focal morphology of ARDS. The relation between re-aeration and improvement in ventilation perfusion mismatch and heart function was not evaluated in this review (Karbing et al., 2020). Furthermore, in this review, we investigated the imaging techniques' role in predicting RM effects in deeply sedated patients without considering the optimal level of PEEP that would be required after recruitment to keep the lung open. Rather than a final classification, we suggest that the morphological classification is a good starting point to further improve from, with the addition of other predictors. Furthermore, more attention should be drawn to the quantification of overdistention rather than measurement of re-aeration alone. Balancing the assessment of negative and positive effects may improve our understanding as to what patients may or may not benefit from RMs.

## Conclusions

We conclude that defining positive response to RMs using imaging techniques is challenging and not yet well-elucidated. Variations in RM method, population selection, as well as different imaging techniques should be taken into consideration in future studies. Given the adverse events associated with high maximum airway pressures, only the lungs of specific patients can be re-aerated with moderate maximum airway pressures. Lung ultrasound and CT characteristics consistent with non-focal morphology of ARDS are predictive of more re-aeration in response to recruitment maneuver. The morphological characteristics related to successful response to RMs in patients without ARDS have not been studied to date.

## Data Availability Statement

The original contributions presented in the study are included in the article/Supplementary Material, further inquiries can be directed to the corresponding author/s.

## Author Contributions

CP performed the literature search, drafted the manuscript, and approved the submitted version of the manuscript. MRS, LH, NH, MH, and FP revised the manuscript for critical content and approved the submitted version of the manuscript. MJS and LB conceived the study, revised the manuscript for critical content, and approved the submitted version of the manuscript. All authors contributed to the article and approved the submitted version.

## Conflict of Interest

The authors declare that the research was conducted in the absence of any commercial or financial relationships that could be construed as a potential conflict of interest.
