# Supplementary material for: Assessment of the Effect of Recruitment Maneuver on Lung Aeration Through Imaging Analysis in Invasively Ventilated Patients: A Systematic Review
Source: Front Physiol. 2021 Jun 4;12:666941. doi: 10.3389/fphys.2021.666941 (PMC8212037; doi:10.3389/fphys.2021.666941)
Supplement: Supplementary file 1 [file Table_1.DOCX]

**Assessment of the effect of recruitment maneuver on lung aeration through imaging analysis in invasively ventilated patients: a systematic review.**

Authors:

Charalampos Pierrakos (1,5), Marry R Smit (1), Laura A Hagens (1), Nanon F L Heijnen (6), Markus W Hollmann (2-3), Marcus J Schultz (1, 7-8), Frederique Paulus (1), Lieuwe D.J. Bos (1,2, 4)

**Amsterdam UMC, location AMC, University of Amsterdam, Amsterdam, The Netherlands:**

^1^ Department of Intensive Care

^2^ Laboratory of Experimental Intensive Care and Anesthesiology

^3^ Department of Anesthesiology

^4^ Department of Respiratory Medicine

**Brugmann University Hospital, Université Libre de Bruxelles,Brussels, Belgium:**

^5^ Department of Intensive Care

**Maastricht UMC+, Maastricht, The Netherlands**

^6^ Department of Intensive Care

**Mahidol University, Bangkok, Thailand:**

^7^ Mahidol–Oxford Tropical Medicine Research Unit (MORU)

**University of Oxford, Oxford, UK:**

^8^ Nuffield Department of Medicine

Supplement

|  | Patient selection | | Index test | | Reference standard | | Flow and Timing |
| --- | --- | --- | --- | --- | --- | --- | --- |
|  | Risk of Bias | Applicability | Risk of Bias | Applicability | Risk of Bias | Applicability | Risk of bias |
| LUS studies |  |  |  |  |  |  |  |
| Genereux et al 2019 [1] | Low | Low | Low | Low | Unclear | Unclear | Low |
| Tang et al 2017 [2] | Unclear | Low | Low | Low | Unclear | Unclear | Low |
| Longo et al 2017 [3] | Low | High | Low | Low | Unclear | Unclear | Unclear |
| Rode et al 2012 [4] | Unclear | Low | Low | Low | Unclear | Unclear | Low |
| Bouhemad et al 2011 [5] | Unclear | Low | Low | Low | Unclear | Unclear | Low |
| EIT studies |  |  |  |  |  |  |  |
| He et al 2020 [6] | Low | Low | Low | Low | Unclear | Unclear | Low |
| Karsten et al 2019 [7] | Unclear | Low | Low | Low | Unclear | Unclear | Low |
| Zhao et al 2019 [8] | High | Low | High | High | Unclear | Unclear | Low |
| Eichler et al 2018 [9] | Low | High | Low | Unclear | Unclear | Unclear | Low |
| Eronia et al 2017 [10] | Unclear | Low | Low | Low | Unclear | Unclear | Low |
| CT studies |  |  |  |  |  |  |  |
| Camporota et al 2019 [11] | Low | Low | Low | Low | Unclear | Low | Low |
| Chiumello et al 2016 [12] | Unclear | Low | Low | Low | Low | Low | Low |
| Caironi et al 2015 [13] | High | Low | Low | Low | Low | Low | Low |
| de Matos et al 2012 [14] | Low | Low | Low | Low | Low | Low | Low |
| Constantin et al 2010 [15] | Low | Low | Low | Low | Low | Low | Low |
| Caironi et al 2010 [16] | Low | Low | Low | Low | Unclear | Low | Low |
| Gattinoni et al 2006 [17] | Low | Low | Low | Low | Low | Low | Low |
| Borges et al 2006 [18] | Low | Low | Low | Low | Low | Low | Low |
| Nieszhowska et al 2004 [19] | High | Low | Low | Low | Low | Low | Low |
| Vieira et al 1999 [20] | Unclear | Low | Low | Low | Low | Low | Low |

**References**

1. Généreux V, Chassé M, Girard F, Massicotte N, Chartrand-Lefebvre C, Girard M. Effects of positive end-expiratory pressure/recruitment manoeuvres compared with zero end-expiratory pressure on atelectasis during open gynaecological surgery as assessed by ultrasonography: a randomised controlled trial. *Br J Anaesth*. 2020 Jan;124(1):101–9.

2. Tang KQ, Yang SL, Zhang B, Liu HX, Ye DY, Zhang HZ, et al. Ultrasonic monitoring in the assessment of pulmonary recruitment and the best positive end-expiratory pressure. *Medicine (Baltimore)*. 2017;96(39):e8168.

3. Longo S, Siri J, Acosta C, Palencia A, Echegaray A, Chiotti I, et al. Lung recruitment improves right ventricular performance after cardiopulmonary bypass A randomised controlled trial. *Eur J Anaesthesiol*. 2017;34(2):66–74.

4. Rode B, Vučić M, Širanović M, Horvat A, Krolo H, Kelečić M, et al. Positive end-expiratory pressure lung recruitment: Comparison between lower inflection point and ultrasound assessment. *Wien Klin Wochenschr*. 2012;124(23–24):842–7.

5. Bouhemad B, Brisson H, Le-Guen M, Arbelot C, Lu Q, Rouby JJ. Bedside ultrasound assessment of positive end-expiratory pressure-induced lung recruitment. *Am J Respir Crit Care Med*. 2011;183(3):341–7.

6. He H, Chi Y, Long Y, Yuan S, Frerichs I, Möller K, Fu F, Zhao Z. Influence of overdistension/recruitment induced by high positive end-expiratory pressure on ventilation-perfusion matching assessed by electrical impedance tomography with saline bolus. Crit Care. 2020 Sep 29;24(1):586.

7. Karsten J, Voigt N, Gillmann H-J, Stueber T. Determination of optimal positive end-expiratory pressure based on respiratory compliance and electrical impedance tomography: a pilot clinical comparative trial. *Biomed Eng / Biomed Tech*. 2019 Apr 24;64(2):135–45.

8. Zhao Z, Lee LC, Chang MY, Frerichs I, Chang HT, Gow CH, et al. The incidence and interpretation of large differences in EIT-based measures for PEEP titration in ARDS patients. *J Clin Monit Comput.* 2019;10.1007/s10877-019-00396-8.

9. Eichler L, Truskowska K, Dupree A, Busch P, Goetz AE, Zöllner C. Intraoperative Ventilation of Morbidly Obese Patients Guided by Transpulmonary Pressure. *Obes Surg*. 2018;28(1):122–9.

10. Eronia N, Mauri T, Maffezzini E, Gatti S, Bronco A, Alban L, et al. Bedside selection of positive end-expiratory pressure by electrical impedance tomography in hypoxemic patients: a feasibility study. *Ann Intensive Care*. 2017 Dec 20;7(1):76.

11. Camporota L, Caricola E V., Bartolomeo N, Di Mussi R, Wyncoll DLA, Meadows CIS, et al. Lung Recruitability in Severe Acute Respiratory Distress Syndrome Requiring Extracorporeal Membrane Oxygenation. *Crit Care Med*. 2019;47(9):1177–83.

12. Chiumello D, Marino A, Brioni M, Cigada I, Menga F, Colombo A, et al. Lung recruitment assessed by respiratory mechanics and computed tomography in patients with acute respiratory distress syndrome what is the relationship? *Am J Respir Crit Care Med*. 2016;193(11):1254–63.

13. Caironi P, Carlesso E, Cressoni M, Chiumello D, Moerer O, Chiurazzi C, et al. Lung recruitability is better estimated according to the Berlin definition of acute respiratory distress syndrome at standard 5 cm H2O rather than higher positive end-expiratory pressure: a retrospective cohort study. *Crit Care Med*. 2015;43(4):781–90.

14. de Matos GFJ, Stanzani F, Passos RH, Fontana MF, Albaladejo R, Caserta RE, et al. How large is the lung recruitability in early acute respiratory distress syndrome: A prospective case series of patients monitored by computed tomography. *Crit Care*. 2012;16(1):R4.

15. Constantin JM, Grasso S, Chanques G, Aufort S, Futier E, Sebbane M, et al. Lung morphology predicts response to recruitment maneuver in patients with acute respiratory distress syndrome. *Crit Care Med*. 2010;38(4):1108–17.

16. Caironi P, Cressoni M, Chiumello D, Ranieri M, Quintel M, Russo SG, et al. Lung opening and closing during ventilation of acute respiratory distress syndrome. *Am J Respir Crit Care Med*. 2010;181(6):578–86.

17. Gattinoni L, Caironi P, Cressoni M, Chiumello D, Ranieri VM, Quintel M, et al. Lung recruitment in patients with the acute respiratory distress syndrome. *N Engl J Med*. 2006;354(17):1775–86.

18. Borges JB, Okamoto VN, Matos GFJ, Caramez MPR, Arantes PR, Barros F, et al. Reversibility of lung collapse and hypoxemia in early acute respiratory distress syndrome. *Am J Respir Crit Care Med*. 2006;174(3):268–78.

19. Nieszkowska A, Lu Q, Vieira S, Elman M, Fetita C, Rouby JJ. Incidence and regional distribution of lung overinflation during mechanical ventilation with positive end-expiratory pressure. *Crit Care Med*. 2004;32(7):1496–503.

20. Vieira SRR, Puybasset L, Lu Q, Richecoeur J, Cluzel P, Coriat P, et al. A scanographic assessment of pulmonary morphology in acute lung injury: Significance of the lower inflection point detected on the lung pressure- volume curve. *Am J Respir Crit Care Med*. 1999;159(5 I):1612–23.
